# Autogenous Translational Regulation of the Borna Disease Virus Negative Control Factor X from Polycistronic mRNA Using Host RNA Helicases

**DOI:** 10.1371/journal.ppat.1000654

**Published:** 2009-11-06

**Authors:** Yohei Watanabe, Naohiro Ohtaki, Yohei Hayashi, Kazuyoshi Ikuta, Keizo Tomonaga

**Affiliations:** 1 Department of Virology, Research Institute for Microbial Diseases (BIKEN), Osaka University, Suita, Osaka, Japan; 2 Section of Viral Infections, Thailand–Japan Research Collaboration Center on Emerging and Re-emerging Infections (RCC-ERI), Nonthaburi, Thailand; 3 PRESTO, Japan Science and Technology Agency (JST), Chiyoda-ku, Tokyo, Japan; Mount Sinai School of Medicine, United States of America

## Abstract

Borna disease virus (BDV) is a nonsegmented, negative-strand RNA virus that employs several unique strategies for gene expression. The shortest transcript of BDV, X/P mRNA, encodes at least three open reading frames (ORFs): upstream ORF (uORF), X, and P in the 5′ to 3′ direction. The X is a negative regulator of viral polymerase activity, while the P phosphoprotein is a necessary cofactor of the polymerase complex, suggesting that the translation of X is controlled rigorously, depending on viral replication. However, the translation mechanism used by the X/P polycistronic mRNA has not been determined in detail. Here we demonstrate that the X/P mRNA autogenously regulates the translation of X via interaction with host factors. Transient transfection of cDNA clones corresponding to the X/P mRNA revealed that the X ORF is translated predominantly by uORF-termination-coupled reinitiation, the efficiency of which is upregulated by expression of P. We found that P may enhance ribosomal reinitiation at the X ORF by inhibition of the interaction of the DEAD-box RNA helicase DDX21 with the 5′ untranslated region of X/P mRNA, via interference with its phosphorylation. Our results not only demonstrate a unique translational control of viral regulatory protein, but also elucidate a previously unknown mechanism of regulation of polycistronic mRNA translation using RNA helicases.

## Author Summary

All viruses rely on host cell factors to complete their life cycles. Therefore, the replication strategies of viruses may provide not only the understanding of virus pathogenesis but also useful models to disentangle the complex machinery of host cells. Translation regulation of viral mRNA is a good example of this. Borna disease virus (BDV) is a highly neurotropic RNA virus which is characterized by persistent infection. BDV expresses mRNAs as polycistronic coding transcripts. Among them, the 0.8 kb X/P mRNA encodes at least three open reading frames (ORFs), upstream ORF, X, and P. Although BDV X and P have opposing effects in terms of viral polymerase activity, the translational regulation of X/P polycistronic mRNA has not been elucidated. In this study, we show an ingenious strategy of translational control of viral regulatory protein using host factors. We demonstrate that host RNA helicases, mainly DDX21, can affect ribosomal reinitiation of X via interaction with the 5′ untranslated region (UTR) of X/P mRNA and that the downstream P protein autogenously controls the translation of X by interfering with the binding of DDX21 to the 5′ UTR. Our findings uncover not only a unique translational control of viral regulatory protein but also a previously unknown mechanism of translational regulation of polycistronic mRNA using RNA helicases.

## Introduction

The control of translation initiation on mRNA is one of the most fundamental processes in the regulation of gene expression. Most eukaryotic mRNAs initiate translation via the so-called “scanning mechanism”, in which the 40S ribosomal subunit binds to the cap structure at the 5′-terminus of mRNA and slides to the proximal AUG codon [1]. In this mechanism, translation initiation from the downstream AUGs is generally inefficient. Thus, the eukaryotic cellular genes are transcribed individually, generating monocistronic mRNAs. On the other hand, many animal viruses produce polycistronic mRNAs and express efficiently functionally different proteins from a single mRNA molecule [2]–[5], suggesting that eukaryotic ribosomes have the potential to initiate the translation of downstream ORFs, under the control of sequence- and/or structure-dependent features of the mRNAs.

Polycistronic coding by mRNAs is a means of coordinating the expression of more than two proteins, which are arranged in tandem or overlapping in a single mRNA molecule [6],[7]. Analysis of polycistronic mRNAs therefore provides a better understanding of the regulatory mechanisms of ribosomal scanning during mRNA translation. In the leaky scanning mechanism, ribosomes bypass the first start codon when the context is poor and thus reach a start codon further downstream. Some viruses, such as Sendai virus and papillomaviruses, use such mechanisms to enable a multifunctional mRNA to express several proteins with different functions in viral replication [8]–[10]. Another strategy for translation of downstream cistrons from an mRNA is termination/reinitiation, is the major method of translation of prokaryotic and some viral mRNAs [11]–[13]. In this case, ribosomes resume the scanning of the mRNA and reinitiate translation efficiently at a downstream AUG codon, following the termination of an upstream cistron. Although eukaryotic ribosomes are in general unable to reinitiate downstream cistrons on an mRNA, it is also true that about 10 to 30% of eukaryotic mRNAs contain upstream AUG codons (uAUG), which have the capacity to initiate translation of a short upstream ORF (uORF), usually consisting of fewer than 30 codons [14]–[16]. The uORF-mediated reinitiation of downstream ORFs also has been demonstrated in eukaryotic mRNAs [17]–[20], suggesting that ribosomal termination/reinitiation may be a key mechanism for the regulation of complex gene expression in eukaryotic cells. However, we know little about the molecular mechanisms underlying the regulation of ribosomal initiation in the translation of polycistronic mRNA, especially how eukaryotic viruses use translational regulation in the expression of viral proteins.

**Figure 1:**
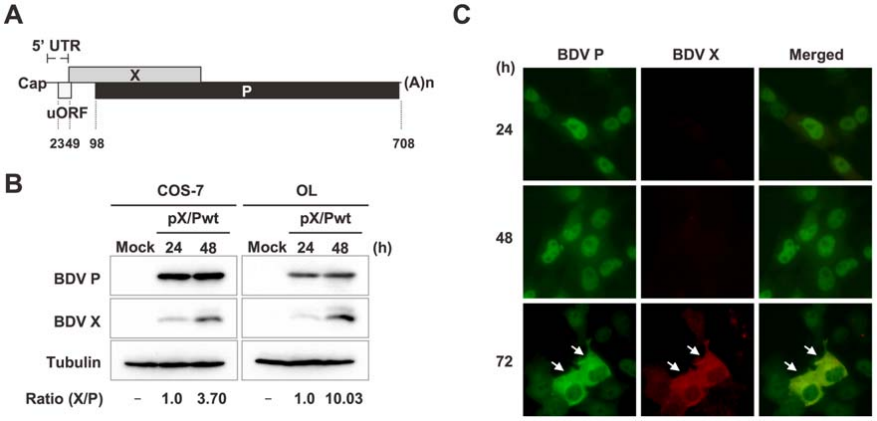
Autogenous regulation of BDV X translation from the X/P polycistronic mRNA. (A) A schematic representation of the 0.8 kb BDV polycistronic X/P mRNA. The numbers indicate nucleotide positions in the X/P mRNA. (B) COS-7 cells and OL cells were transfected with pX/Pwt, and the total cell lysates were used for Western blotting probed with anti-BDV P and X antibodies at 24 and 48 h post-transfection. The expression ratio of X to P was determined after quantitation of band intensities by ImageJ software. (C) OL cells were transfected with pX/Pwt (0.5 µg) and the subcellular localization of X and P was determined by immunofluorescence assay. Arrows indicate cytoplasmic localization of X and P at 72 h post-transfection.

**Figure 2:**
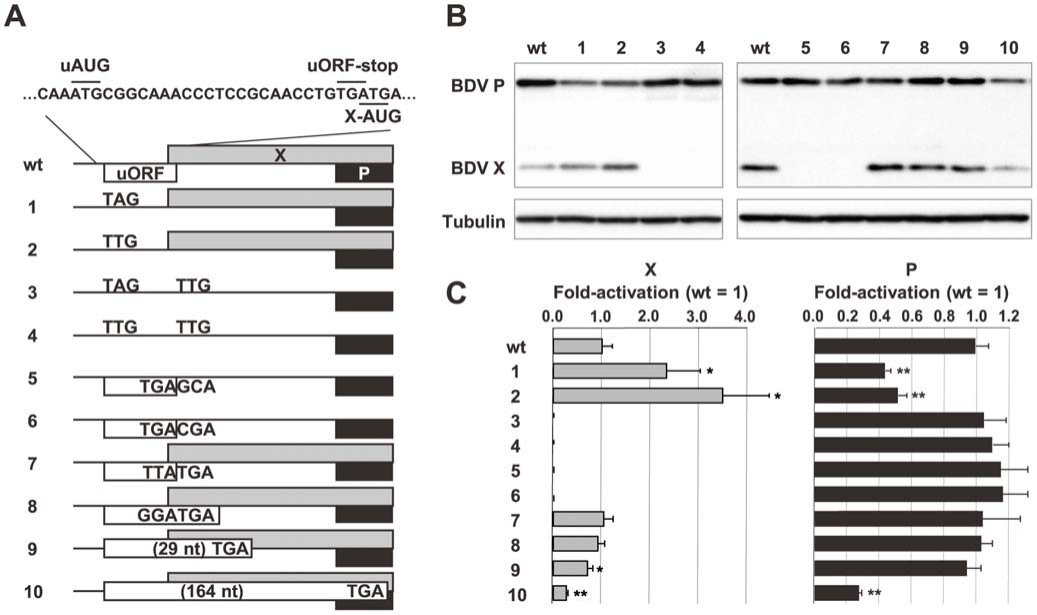
The uORF influences translation of the X and P ORFs. (A) Schematic representation of mutants of the X/P expression plasmid. The nucleotide sequences substituted in the wt plasmid are indicated. (B) OL cells were transfected with 0.8 µg of each plasmid and at 12 h after transfection cells were harvested and subjected to Western blotting using anti-BDV P and X antibodies. (C) Fold-activation of X and P expression in the cells transfected with mutant plasmids was determined after quantitation of band intensities by ImageJ software. The mean plus S.D. of three independent experiments are shown. **P<0.01, *P<0.05 (Student's *t* test).

Borna disease virus (BDV) is a non-segmented, negative-sense RNA virus that belongs to the *Mononegavirales* and which is characterized by highly neurotropic and persistent infection. BDV replicates and is transcribed in the cell nucleus and employs several unique strategies for gene expression [21],[22]. One of the most striking characteristics of this virus is that all of the BDV transcripts have polycistronic coding capacity. The shortest, 0.8 kb transcript of BDV, X/P mRNA, encodes at least three ORFs: uORF, X, and P in the 5′ to 3′ direction (Figure 1A). The X and P ORFs produce major viral proteins, which overlap by 215 nucleotides (nt) [23],[24]. In contrast, the uORF, whose stop codon overlaps the X translation start codon (X-AUG) by one nt, UGAUG, (Figure 2A), encodes an 8 amino acid peptide, the expression of which has not yet been shown in infected cells. BDV X is a negative regulator of viral polymerase activity, while the P phosphoprotein is a necessary cofactor of the polymerase complex [25],[26]. Thus, the expression ratio between X and P is critical for viral polymerase activity [25]–[28]. Previous studies revealed that, despite an optimal sequence context for initiation of X compared to P, translation of X seems to be suppressed at an early stage of viral infection and gradually increases along with the establishment of persistent infection (Figure S1) [29],[30]. This finding allows us to hypothesize that translational regulation of such a short polycistronic mRNA may have evolved to control rigorously the ratio between X and P in the infected cell nucleus and is essential for the maintenance of the persistent infection. Recent studies have suggested that the 5′ untranslated region (5′ UTR) of X/P mRNA plays a critical role in the translational regulation of X from the polycistronic mRNA [28],[31]. However, the translation mechanism used by the X/P polycistronic mRNA has not been determined in detail.

In this study, we demonstrate the autogenous translational regulation of the X/P polycistronic mRNA mediated by host RNA helicases. We show that DDX21, also known as RNA helicase II/Gu, is a regulator of ribosomal reinitiation of X via interaction with the 5′ UTR of X/P mRNA (X/P UTR) and that expression of the downstream P protein may regulate the translation of X by interfering with the binding of DDX21 to the 5′ UTR. Our results provide not only a unique insight into translational control of a viral polycistronic mRNA but also a novel role for RNA helicase in the regulation of ribosomal reinitiation during eukaryotic mRNA translation.

## Results

### Translational regulation of BDV X from the X/P polycistronic mRNA

To investigate translational regulation of the X/P polycistronic mRNA, we first used a plasmid, pX/Pwt [30], which encodes a cDNA clone corresponding to the X/P mRNA, and assessed whether this plasmid is able to reproduce the translational regulation of X/P mRNA independently of BDV infection. Upon transfection into COS-7 and OL cells, the plasmid produced efficiently both X and P, and expression of X appeared to increase following the course of time after transfection (Figure 1B), similar to the expression dynamics of X in BDV-infected cells (Figure S1) [30],[32]. Previous studies revealed that P translocates to the cytoplasm from the nucleus via interaction with X [28],[30],[33]. As shown in Figure 1C, although the cells transfected with pX/Pwt exhibited the nuclear distribution of P at an early time after transfection, P was shown to move to the cytoplasm of the cells expressing X at 72 h post-transfection (arrows). These observations suggested that the X/P mRNA by itself regulates the translation of X independently of BDV infection and, therefore, that pX/Pwt provides a useful tool to investigate the translational regulation of the polycistronic mRNA.

### The uORF controls translation initiation of the X and P ORFs

To understand the role of the uORF in the translation of the X and P ORFs, we generated a series of mutant plasmids (Figure 2A) and examined the expression of X and P by Western blotting at 12 h post-transfection, at which point the expression of X had not yet been upregulated. Mutants with the uAUG replaced by TAG or TTG exhibited markedly increased expression of X compared to the wt plasmid (Figure 2B and 2C, lanes 1 and 2). In addition, the involvement of uORF in the translation of X was demonstrated by using a series of deletion mutants of the 5′ UTR (Figure S2). In contrast, the translation of P was reduced to approximately 50% of the wt plasmid (lanes 1 and 2). In addition, changing the initiation codon of X (X-AUG) to TTG in the above uAUG mutants recovered the expression level of P to the equivalent of the wt plasmid (lanes 3 and 4). Furthermore, single mutants, which lack only the X ORF, with substitution of the X-AUG by AGC or ACG produced P at levels comparable to the wt plasmid with complete abolition of the expression of X (lanes 5 and 6). These observations suggested that the uAUG is recognized efficiently by scanning ribosomes and that the presence of the uORF seems to downregulate the basal expression level of X, while enhancing the translation efficiency of the P ORF to a level equivalent to an mRNA lacking both the uORF and X ORF.

To determine how translation of the X and P ORFs is initiated from the X/P mRNA, we next introduced mutations into the termination codon of the uORF. A mutant in which the uORF stop codon had been changed to TTA expressed both X and P at equivalent levels to the wt plasmid (lane 7). Furthermore, a 3-nt downstream extension of the uORF termination codon also appeared not to influence the translation of either protein (lane 8). Meanwhile, downstream extension of the uORF to 29 nt reduced the expression of X, but not P, to 70% of the wt level (lane 9). Interestingly, a 164 nt extension of the uORF, so that a stop codon is introduced within the P ORF, showed significant decreases (∼30%) in the expression of both X and P (lane 10). The introduction of premature stop codons within the uORF also reduced the expression level of X (Figure S3). These results indicated that the presence of the uORF termination codon in close proximity to the X-AUG seems to be important for efficient translation of the X ORF. Furthermore, termination of the uORF before initiation of the P ORF is required for the expression of P. We also demonstrated that P is unlikely to be expressed by ribosomal shunting or internal ribosome entry site-mediated mechanism (Figure S4). Taken together, both the X and P ORFs were shown to be translated predominantly by ribosomal reinitiation, dependent on uORF termination, and the remainder of the translation (approximately 30%) might be initiated by leaky scanning of upstream start codons.

### Translation initiation of the X ORF is upregulated by P

The results shown above suggest that uORF-termination/reinitiation may play a key role in the regulation of translation of X. Therefore, we sought next to investigate the factors that influence ribosomal reinitiation at the X ORF. At first, we examined the role of the peptide predicted to be produced by the uORF. However, we could not detect any effects of this predicted peptide on the translation of the downstream ORFs (Figure S5).

**Figure 3:**
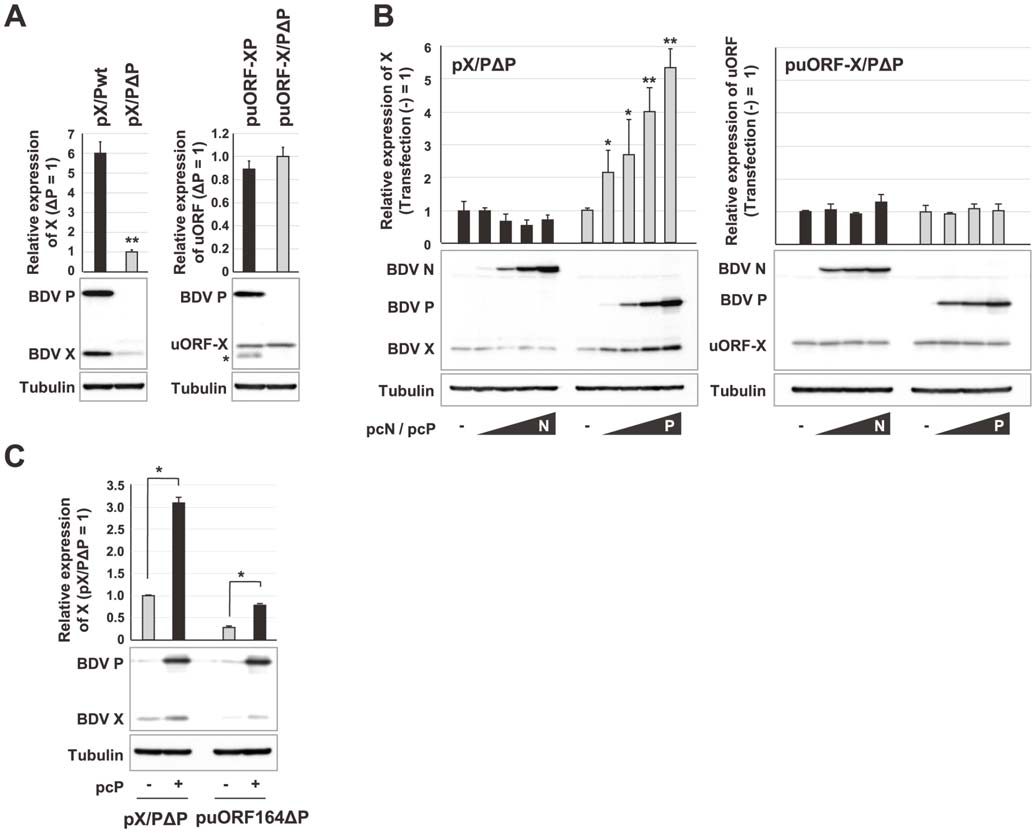
Expression of BDV P enhances translation of the X ORF. (A) OL cells were transfected with 0.8 µg of the plasmids indicated and, 48 h after transfection, expression of the X and uORF-X fusion proteins was detected by Western blotting using anti-BDV P and X antibodies. The asterisk in the puORF-X/P-transfected panel indicates a non-specific reaction. (B) OL cells were cotransfected with pX/PΔP or puORF-X/PΔP (0.4 µg) and a four-fold dilution series of BDV N (pcN) or P (pcP) expression plasmid (0.00625, 0.025, 0.1 and 0.4 µg). The empty plasmid was used for equilibration of the total amount of transfected plasmid. The cells were harvested at 48 h post-transfection and subjected to Western blotting. The expression of BDV N was detected by anti-BDV N monoclonal antibody. (C) OL cells were transfected with 0.8 µg of the plasmids indicated in the presence or absence of P and, 48 h after transfection, expression of the P and X was detected by Western blotting using anti-BDV P and X antibodies. Relative expression levels of detected proteins were determined after quantitation of band intensities by ImageJ software. The mean plus S.D. of three independent experiments are shown. **P<0.01, *P<0.05 (Student's *t* test).

We next examined the effect of the protein encoded downstream, P, because this may accumulate in the nucleus in association with the expression of the X/P mRNA [34]. We generated mutant plasmids, pX/PΔP, in which the initiation codon of P, P-AUG, was substituted by TTG, and puORF-X/PΔP, in which the uORF was fused in-frame to the X ORF in pX/PΔP, and assessed the production of X at 48 h post-transfection, at which point P should have accumulated sufficiently in wt plasmid-transfected cells. As shown in Figure 3A, translation of X was significantly higher in cells transfected with pX/Pwt than with pX/PΔP. In contrast, initiation of translation of the uORF-X fusion protein appeared not to be affected by deletion of the P-AUG. These results indicated that expression of P upregulates the translation of the X ORF without affecting the ribosomal initiation from the uAUG. To verify this, a plasmid expressing P, pcP [33], was cotransfected with pX/PΔP or puORF-X/PΔP. Interestingly, expression of X, but not the uORF-X fusion protein, was enhanced markedly in a dose-dependent manner by co-expression of P, while the nucleoprotein (N) of BDV failed to enhance the expression of X in transfected cells (Figure 3B). A ΔP mutant based on a 164-bp uORF-extension plasmid (Figure 2A, lane 10), puORF164ΔP, also upregulated the expression of X in the presence of P (Figure 3C), although the basal expression level of X in the puORF164ΔP is significantly lower than that in pX/PΔP both with or without P. These data suggested that P influences the ribosomal initiation at the X-AUG predominantly by the uORF-termination-coupled reinitiation mechanism, while a leaky scanning mechanism may be involved to some extent in the upregulation of the translation of X by P.

### Nuclear factor(s) inhibit translation of the X ORF by binding to the 5′ UTR

We set out to address next the question of how the expression of P can enhance the reinitiation of translation of X. First, we investigated the possibility that P may interact directly with the X/P mRNA and then influence ribosomal reinitiation at the X ORF. We could not demonstrate, however, a direct interaction between P and the X/P mRNA by immunoprecipitation (IP)-RT-PCR analysis (data not shown). Second, it might be possible that P affects the functions of eukaryotic initiation factors (eIFs), such as eIF2α and eIF3. However, an interaction between eIFs and BDV P was not demonstrable in cells transfected with BDV P (Figure S6A). Furthermore, expression of eIF2α, 2Bε and 3A, as well as the serine phosphorylation level of eIF2α, appeared not to be changed in cells expressing P (Figure S6B), indicating that the expression of P is unlikely to affect the quantitative and qualitative properties of eIFs.

**Figure 4:**
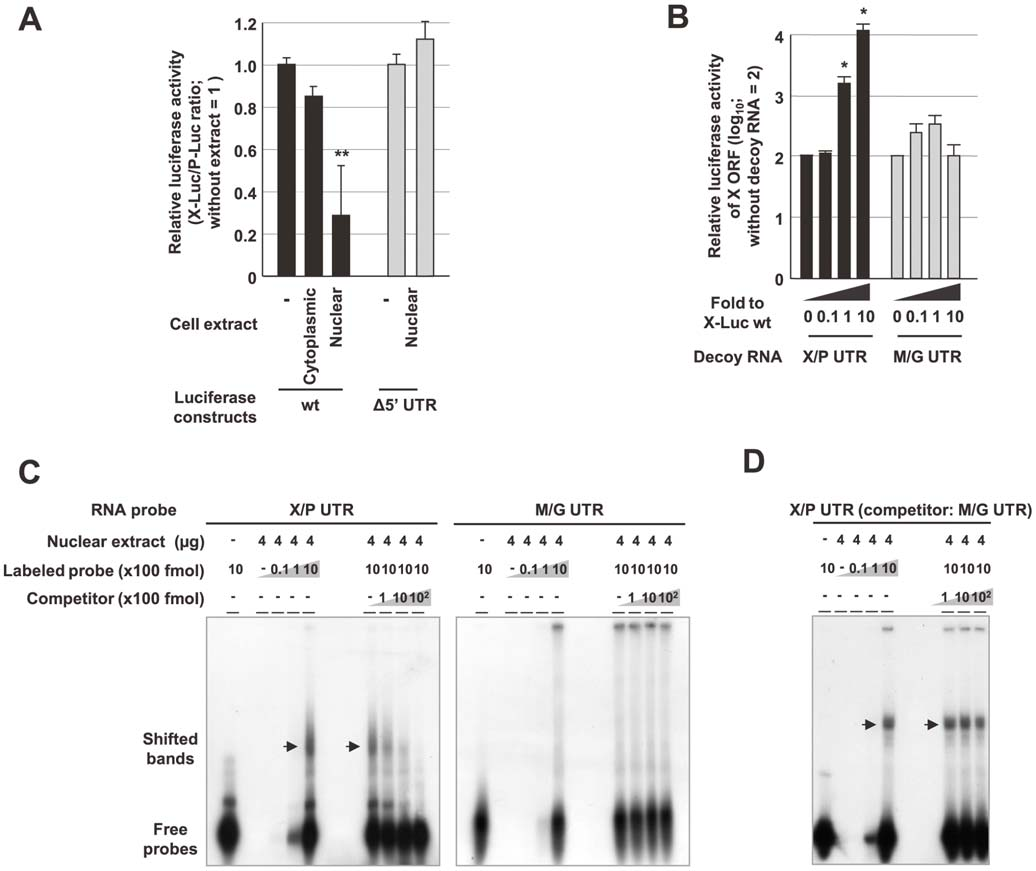
Nuclear proteins suppress translation of the X ORF via the 5′ UTR of the mRNA. (A) A nuclear extract inhibits the initiation of translation of the X ORF. The extracts (1 µg) from OL cells were incubated with *in vitro* transcribed X- or P-Luciferase fusion RNA (X-Luc, P-Luc) in 20 µl of binding buffer. After the reaction, *in vitro* translation was performed using a reticulocyte lysate mixture. After the incubation, 10 µl of the mixture was subjected to luciferase assay. Δ5′ UTR indicates luciferase plasmids lacking the 5′ UTR of X/P mRNA. (B) The nuclear factors interact with the X/P UTR. A serial amount of decoy RNA (X/P and M/G UTR) was incubated with the nuclear extract (4 µg) prior to incubation with X-Luc RNA, and then *in vitro* translation was performed using the reticulocyte lysate mixture. Relative luciferase activities of the X fusion protein were determined. The mean plus S.D. of three independent experiments are shown. **P<0.01, *P<0.05 (Student's *t* test). (C and D) RNA EMSAs were performed using nuclear extracts of OL cells as described in Methods. For competition, serial amounts of non-labeled own (C) or M/G UTR probes (D) were incubated with the nuclear extract. Arrows indicate shifted bands produced by incubation with the nuclear extract. Bound complexes were resolved from free RNA by electrophoresis in 4% native polyacrylamide gels.

We therefore considered the possibility that P enhances the translation of X indirectly by interaction with cellular factor(s), which may affect ribosomal reinitiation at the X ORF. To investigate this, we conducted first an *in vitro* translation assay using *in vitro* transcribed X/P mRNAs encoding firefly luciferase fused with the X ORF and cellular extracts of OL cells. As shown in Figure 4A, luciferase activity was markedly reduced in the presence of the nuclear extract, but not the cytoplasmic extract, demonstrating that the nucleus may contain factor(s) that suppress translation initiation from the X-AUG. Interestingly, a mutant X/P RNA, which lacks a 38-mer from the 5′ end of the UTR, retained luciferase activity even in the presence of the nuclear extract (Figure 4A), suggesting that nuclear factors may influence the translation of X via interaction with the 5′ UTR. To verify this, we used 48-mer decoy RNAs, which represent the X/P UTR and, as a control, the 5′ UTR of another BDV polycistronic mRNA (M/G UTR). As shown in Figure 4B, incubation with the X/P UTR decoy RNA, but not the M/G UTR control, interfered with the inhibitory effect of the nuclear extract in a dose-dependent manner. We also performed an RNA-electrophoretic mobility shift assay (RNA-EMSA) using ^32^P-labeled riboprobes corresponding to the X/P UTR and M/G UTR to determine whether the nuclear extract interacts specifically with the X/P UTR. As shown in Figure 4C, we found that the X/P UTR riboprobe forms complexes with the nuclear extract (arrows) and that an excess of cold riboprobe efficiently interferes with complex formation. On the other hand, the M/G UTR probe failed not only to generate clear complexes with the extract (Figure 4C) but also to interfere with the complex formation of X/P UTR probe (Figure 4D). All these observations suggested the presence of nuclear factors that inhibit the translation of the X ORF via interaction with the 5′ UTR of X/P mRNA.

### Identification of the 5′ UTR-binding proteins of X/P mRNA

**Figure 5:**
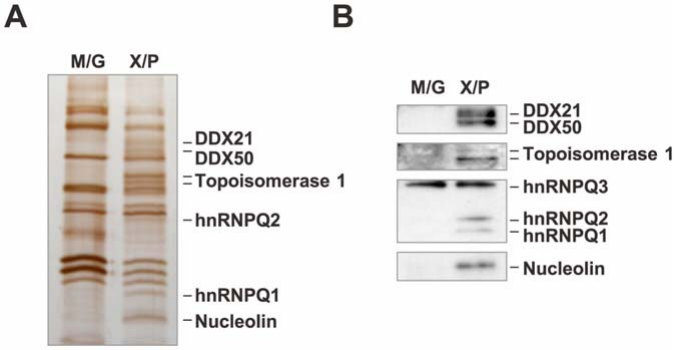
Identification of the 5′ UTR-binding proteins. (A) Silver staining of sequential RNA column purified proteins is shown. The bands specific for the X/P UTR probe (X/P) are indicated. M/G represents a control RNA column using the 5′ UTR of M/G mRNA. (B) The specificity of identified proteins was determined by Western blotting using antibodies specific to each protein (see Materials and Methods).

To investigate in more detail the involvement of nuclear factors in the translational regulation of X, we tried to identify the nuclear factors using a stepwise purification assay and RNA-affinity columns coupled with short (20 mer) and full-length (48 mer) X/P UTR RNAs (see Materials and Methods). The nonspecific RNA binding was visualized using an RNA-affinity column coupled with M/G UTR control RNA. As shown in Figure 5A, seven specific bands were detected by SDS-PAGE. The bands were excised and digested with trypsin and analyzed further by LC-MS/MS. We found that these bands represent DDX21, DDX50, DNA topoisomerase 1 (TOP1), hnRNPQ1/2 and nucleolin (Figure 5A). The accuracy of this analysis was confirmed by Western blotting with antibodies specific for each protein (Figure 5B).

**Figure 6:**
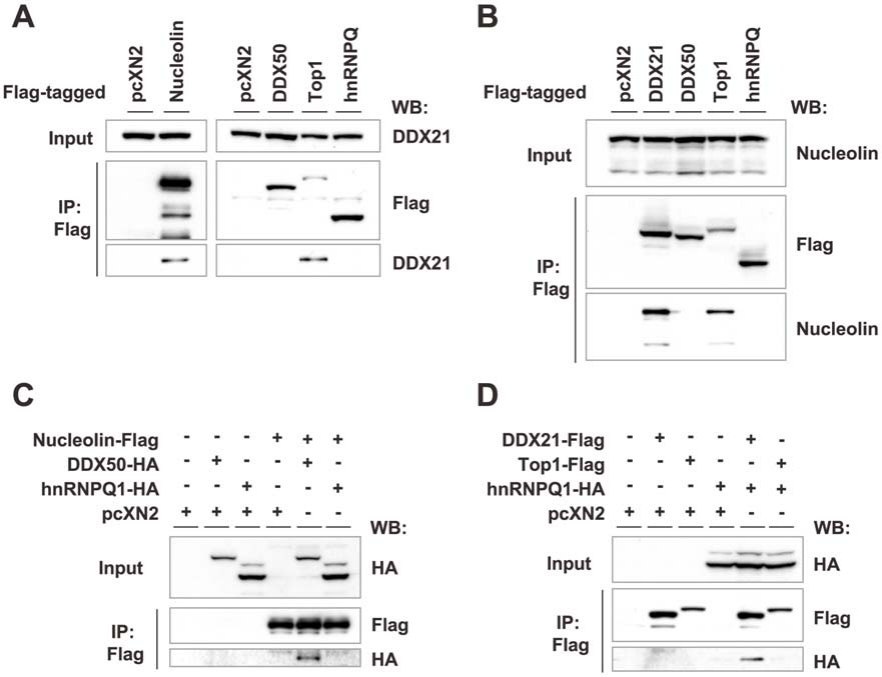
Interaction among the 5′ UTR-binding proteins. (A and B) Interaction of endogenous DDX21 (A) and nucleolin (B) with Flag-tagged UBPs. OL cells were transfected with 10 µg of each Flag-tagged UBP expression plasmid and lysates were immunoprecipitated with anti-FLAG antibody. Western blot analysis was performed using anti-Flag and anti-DDX21 (A) or anti-nucleolin (B) antibodies. (C and D) Pull-down analyses of Flag- and HA-tagged UBPs. OL cells were co-transfected with a combination of 5 µg each of the Flag-tagged and HA-tagged UBPs expression plasmids indicated. At 36 h post-transfection, cell lysates were immunoprecipitated with anti-FLAG antibody. Western blot analysis was performed using anti-HA antibody.

Among the X/P UTR-binding proteins (UBPs), direct interaction has been demonstrated only between nucleolin and TOP1 [35]. Coimmunoprecipitation experiments using Flag-tagged UBPs revealed interactions among DDX21, nucleolin, and TOP1 (Figure 6A and 6B). In addition, interactions of DDX50 and hnRNPQ1 with nucleolin and DDX21, respectively, were demonstrated when hemagglutinin (HA)-tagged proteins were expressed as targets (Figure 6C and 6D). Furthermore, we demonstrated that nucleolin interacts with DDX21 through its C-terminal region using a pull-down analysis with GST- and His-fused recombinant proteins (Figure S7). These observations indicated that the UBPs interact with each other and may have been isolated from the affinity columns as complexes.

**Figure 7:**
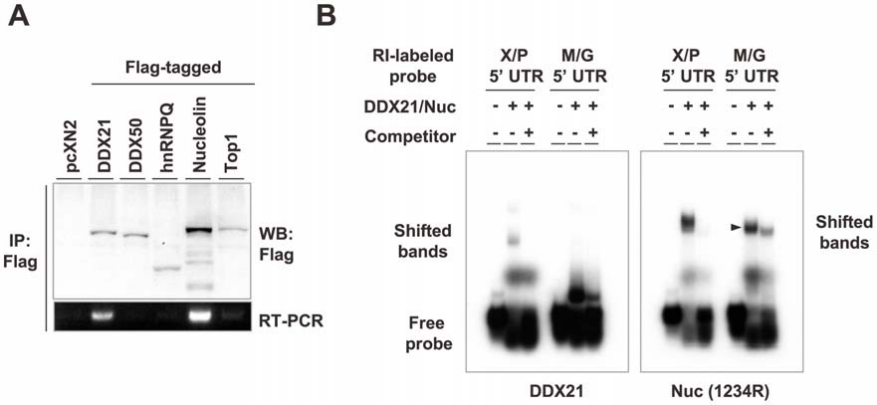
DDX21 is a core protein interacting with the 5′ UTR of X/P mRNA. (A) Immunoprecipitation RT-PCR analysis of UBPs in BDV-infected cells. BDV-infected OL cells were transfected with Flag-tagged UBP expression plasmids. Cell lysates were prepared with RIPA buffer including ribonuclease inhibitor and immunoprecipitated with anti-Flag antibody. The co-purified RNAs in the immunoprecipitates were recovered in TE buffer as described in the Methods section and RT-PCR analysis was performed using a specific primer set for X/P mRNA. (B) RNA EMSA was performed by incubating ^32^P-labelled X/P UTR or M/G UTR riboprobe with GST-tagged DDX21 or nucleolin [Nuc (1234R)]. For competition, non-labeled probes were incubated with each recombinant protein. Bound complexes were resolved from free RNA by electrophoresis in 4% native polyacrylamide gels.

To determine which UBPs contribute dominantly in binding to the X/P UTR, we performed IP-RT-PCR analysis using BDV-infected OL cells and X/P mRNA-specific primers. As shown in Figure 7A, X/P mRNA was amplified clearly from the cells transfected with Flag-tagged DDX21 and nucleolin. Furthermore, RNA EMSA using GST-fused DDX21 and nucleolin revealed that DDX21 interacts directly only with the X/P UTR (Figure 7B), while nucleolin binds to both the X/P UTR and the control M/G UTR (arrowhead). We also found by competitive EMSA that DDX21 binds more strongly to the X/P UTR than nucleolin (data not shown). Nucleolin binds a wide variety of DNA/RNA molecules and is known usually to work in concert with other proteins, which may provide the functional specificity [36],[37]. Along with these properties of nucleolin, our results strongly suggested that DDX21 is a core protein that interacts with the X/P UTR.

### DDX21 causes structural alteration of the X/P UTR

**Figure 8:**
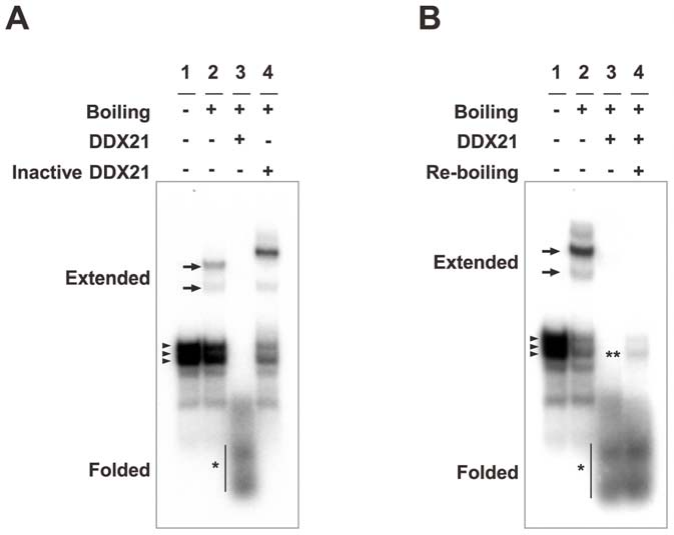
DDX21 causes structural alterations of X/P UTR. (A and B) *In vitro* RNA folding assays were performed with 1.0 pmol of ^32^P-labeled X/P UTR riboprobe. The labeled riboprobe was incubated with 5 pmol of GST-tagged DDX21 and the folding reactions were detected as described in the Methods section. After the incubation, the reaction mixtures were applied to a 12% native polyacrylamide gel. After electrophoresis, the gel was exposed to X-ray film overnight at −80°C. Arrowheads indicate the X/P UTR riboprobe without boiling. Arrows and asterisks represent the extended and folded RNAs on native gels, respectively. Double-asterisks in panel (B) indicate the migration of the riboprobes restored by the re-boiling of the reaction mixture after incubation with DDX21.

DDX21 is known to have RNA helicase activity, which may link to RNA-folding and/or -unwinding through its binding directly to target RNA elements [38]. These observations led us to hypothesize that the interaction of DDX21, along with other UBPs, causes a structural change of the X/P UTR, impacting on ribosomal reinitiation at the X ORF. To determine whether DDX21 alters the structure of the 5′ UTR, therefore, we performed an *in vitro* RNA folding assay using a ^32^P-labeled X/P UTR probe. At first, we monitored the mobility of the X/P UTR riboprobe in a 12% native PAGE with or without boiling. As shown in Figure 8A and 8B, the probe produced low mobility bands after boiling and quick-cooling (lane 2, arrows), in addition to major bands (arrowheads), which are also seen in the gel without boiling (lane 1). The intensities of these low mobility bands were relatively stable during the equilibration period after the quick-cooling step (data not shown), indicating that the low mobility bands represent the extended form of X/P UTR RNA.

To determine the effect of DDX21 on X/P UTR structure, we added GST-fused DDX21 to the RNA probe during the equilibration period. When the X/P UTR probe was reacted with the active DDX21, high mobility bands were observed in the gels (Figure 8A and 8B, asterisks). On the other hand, the heat-inactivated DDX21 failed to produce such bands (Figure 8A). The major bands were restored even in the presence of proteins, when the re-boiling process was conducted after the equilibration step (Figure 8B, double-asterisks). These results suggested that DDX21 caused the folding of the UTR probe and produce the high mobility bands in the gels. Therefore, DDX21 is likely to cause structural alteration of the 5′ UTR of the X/P mRNA.

### BDV P interferes with the interaction of DDX21 and the X/P UTR

**Figure 9:**
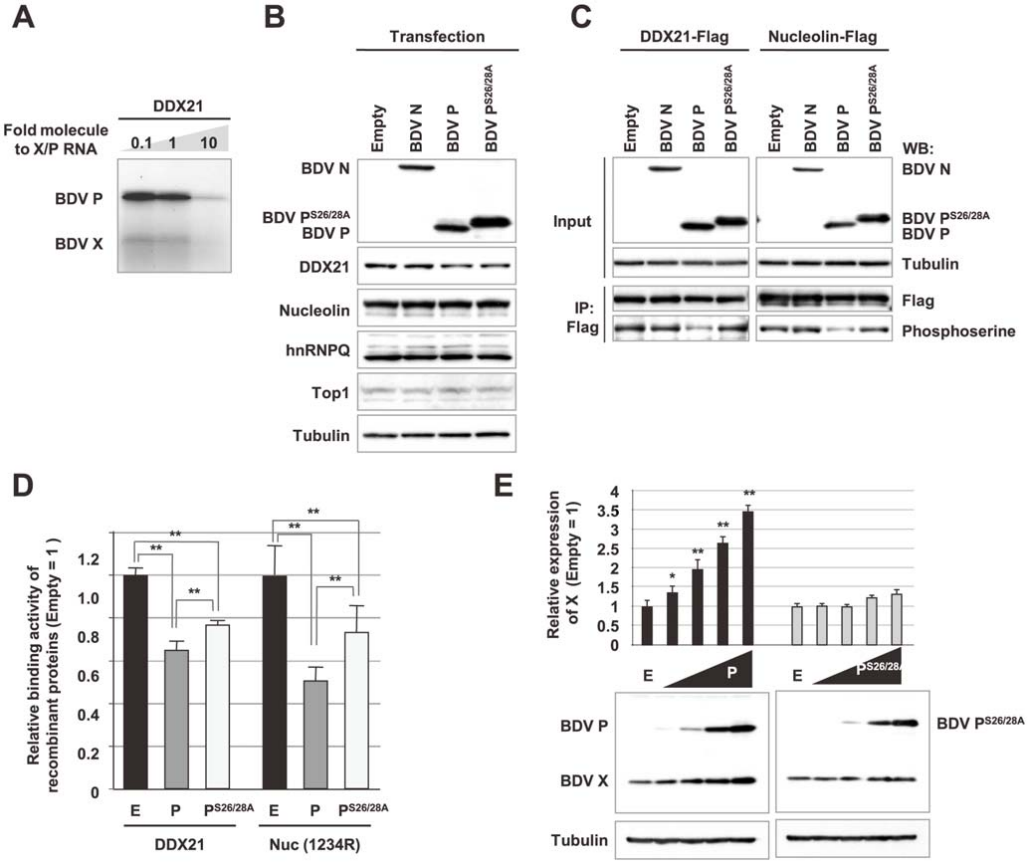
Interaction between BDV P phosphorylation and translation of the X ORF. (A) *In vitro* RNA binding and translation assay of DDX21 and X/P mRNA. *In vitro* transcribed X/P mRNA was incubated with recombinant DDX21 and then *in vitro* translation was performed using a rabbit reticulocyte lysate, according to the manufacturer's recommendations. After incubation, 10 µl of the mixture was subjected to SDS-PAGE and Western blotting using anti-P and -X antibodies. (B) BDV P does not influence the expression of UBPs. OL cells were transfected with plasmids expressing BDV N, P or P^S26/28A^, and 48 h post-transfection the cells were lysed with sample buffer and then subjected to Western blotting using the antibodies indicated. (C) Expression of P reduces phosphorylation levels of DDX21 and nucleolin. Flag-tagged DDX21 or nucleolin was cotransfected with BDV N, P or P^S26/28A^ into OL cells. Forty-eight h after transfection, the cell lysates were immunoprecipitated by anti-Flag antibody and the immunoprecipitants were detected by anti-Flag and anti-phosphoserine antibodies. (D) Expression of P reduces the RNA-binding activity of DDX21 and nucleolin. Flag-tagged recombinant DDX21 and nucleolin were obtained from lysates of OL cells transfected with either empty (E), wt P (P) or mutant P (P^S26/28A^) expression plasmid, and *in vitro* RNA binding assay was performed with ^32^P-labeled X/P UTR riboprobe and purified recombinant proteins as described in the Methods section. Each value represents the mean plus S.D. of at least three independent experiments. **P<0.01, (Student's *t* test). (E) BDV P, but not the P^S26/28A^ mutant, enhances translation of X ORF. OL cells were cotransfected with 0.4 g of pX/PΔP and a serially diluted P or P^S26/28A^ plasmid (4 fold dilution; 0.00625, 0.025, 0.1, 0.4 µg). The expression of BDV X, P and P^S26/28A^ was detected by Western blotting. The relative expression level of X is shown. Each value represents the mean plus S.D. of three independent experiments. E: empty plasmid-transfected. **P<0.01, *P<0.05 (Student's *t* test).

To examine the effect of DDX21 on the translation of the X ORF, we performed a coupled assay of *in vitro* RNA binding and *in vitro* translation using *in vitro* transcribed X/P mRNA and recombinant DDX21. As shown in Figure 9A, incubation with DDX21 reduced the translation not only of X, but also of P, from the X/P mRNA. This result suggested that DDX21 inhibits ribosomal scanning through its binding to the X/P mRNA, resulting in suppression of translation of both X and P. However, the difficulty of the reaction conditions, which must be suitable for *in vitro* RNA binding and *in vitro* translation reactions in the same tube, as well as the possibility that the effect of DDX21 on ribosomal scanning may require its interaction with other UBPs, suggested that this *in vitro* assay is insufficient to determine completely the role of DDX21 in translation. Furthermore, although we generated short-interfering RNAs for UBPs, including DDX21, expression of the siRNAs appeared to induce nonspecific inhibition of the translation of other mRNAs (data not shown). Therefore, we sought to investigate further the effect of DDX21 on the translation of the X ORF by focusing on its interaction with P. P is phosphorylated and acts as a protein kinase substrate, inhibiting the phosphorylation of host proteins to modify their functions [39],[40]. A recent study demonstrated the phosphorylation of DDX21 [41]. Furthermore, the phosphorylation of RNA helicases, such as nucleolin, is known to be critical for RNA-binding activity [42]. Therefore, it is tempting to speculate that interference with phosphorylation by P affects the ability of DDX21 to bind to the X/P UTR. To address this, we examined whether the phosphorylation of DDX21 is affected by the expression of P. OL cells were transfected with wt or mutant P, P^S26/28A^, in which two major phosphorylation sites (Ser26, Ser28) were substituted by alanine [39],[43], and the phosphorylation of DDX21, as well as nucleolin, was monitored. Although the expression levels of the UBPs were unchanged by the expression of P (Figure 9B), the phosphorylation levels of both DDX21 and nucleolin decreased clearly in the cells transfected with wt P, but not with P^S26/28A^ (Figure 9C).

To investigate whether the hypophosphorylation of DDX21 in the presence of P modulates its RNA binding activity, we extracted Flag-tagged DDX21 from the cells transfected with wt P or P^S26/28A^ and then estimated its binding ability to the ^32^P-labelled X/P UTR probe using an *in vitro* RNA binding assay. As shown in Figure 9D, Flag-tagged DDX21, as well as nucleolin, from wt P-transfected cells exhibited significant reduction of binding to X/P UTR. The binding activities of the tagged proteins from the cells transfected with P^S26/28A^ were significantly higher than those with wt P, suggesting that interference with phosphorylation by P decreases the RNA binding activity of DDX21. Therefore, we finally examined whether phosphorylation of P directly affects translation of the X ORF. Consistent with Figure 3B, the expression of X was significantly upregulated in the pX/PΔP-transfected cells in the presence of wt P in a dose-dependent fashion, whereas the P^S26/28A^ mutant was not able to upregulate the translation of X (Figure 9E). Altogether, these results suggested that BDV P may inhibit the binding of DDX21 to the 5′ UTR by interfering with its phosphorylation, resulting in the upregulation of the ribosomal reinitiation from the X-AUG.

## Discussion

In this study, we demonstrated translational regulation of polycistronic mRNA in a unique animal RNA virus. The BDV X/P polycistronic mRNA encodes three overlapping ORFs within a short, 0.8 kb sequence. We showed that the X and P ORFs are translated predominantly by a reinitiation strategy, following the termination of translation of the uORF, although a leaky scanning mechanism is implicated to some extent in the translational processes. In this study, we employed an RNA polymerase II-controlled vector for expression of the X/P mRNA in transfected cells. We have carefully investigated the expression, as well as the structure, of the transcripts from pX/P plasmid DNAs in each experiment and then verified that our system could recreate the translational regulation of X/P mRNA in BDV-infected cells (data not shown). Currently, two types of reinitiation mechanism have been identified in eukaryotic and viral mRNAs [2],[3],[6],[11],[17],[18]. The first type of mRNAs contain short uORFs (<30 codons) upstream of the main ORFs. In this mechanism, the efficiency of reinitiation is controlled by the length of the uORF and by the intercistronic region, an appropriate distance being necessary for the recharging of reinitiation factors, including eIF2 and Met-tRNAiMet, to the ribosomes. Cellular mRNAs such as C/EBP and AdoMetDC, are representative examples of this type of regulation [17],[44]. In the X/P mRNA, initiation of translation of the P ORF may be mediated by this type of reinitiation mechanism. The scanning ribosomes, which travel continuously on the mRNA after termination of translation of the uORF, must recharge the initiation factors on the intercistronic region between the uORF and P ORF and efficiently initiate translation from the P-AUG. Note that the expression level of P is quite invariant, with or without translation of X, if the uORF is present (Figure 2), indicating that the number of ribosomes, which move continuously along the mRNA after uORF termination, is relatively constant on the X/P mRNA. This may be the mechanism underlying the stable and persistent expression of P in infected cells.

The second type of reinitiation mechanism involves mRNAs containing long 5′ ORFs, which usually encode functional proteins. These mRNAs display only short intercistronic distances between the upstream and downstream ORFs, or even may overlap. It has been shown that efficient reinitiation in this mechanism is determined by the stability/mobility of ribosomal complexes to allow reinitiation at the downstream initiation codon [17],[18]. Among viral mRNAs, segment 7 of influenza B virus [45], the ORF-2 of the M2 gene of respiratory syncytial virus (RSV) [46], and the 3′ terminal ORF (VP2) of feline calicivirus (FCV) [47],[48] represent are examples of this type of reinitiation process. Our experiments revealed that reinitiation of the X ORF may be regulated by this type of mechanism, although the uORF encodes only a short and, probably, non-functional peptide. Interestingly, the uORF and X ORF feature an overlapping stop-start codon, UGAUG, as found in other viral polycistronic mRNAs [47],[49],[50]. This feature indicates that the overlapping stop-start codon of the X/P mRNA may play a key role in the regulation of translation of the X ORF. Previous studies revealed that genes divided by such an overlapping stop-start codon are expressed predominantly by termination-coupled translation, in which translation of the downstream ORF is initiated by ribosomes which have read the uORF and stalled at the overlapping stop-start codon [48],[51]. The downstream extension of the termination signal of the uORF in the X/P mRNA significantly reduced the expression of X, suggesting that ribosomal reinitiation from the X-AUG is also carried out by the coupled translation mechanism associated with uORF termination.

**Figure 10:**
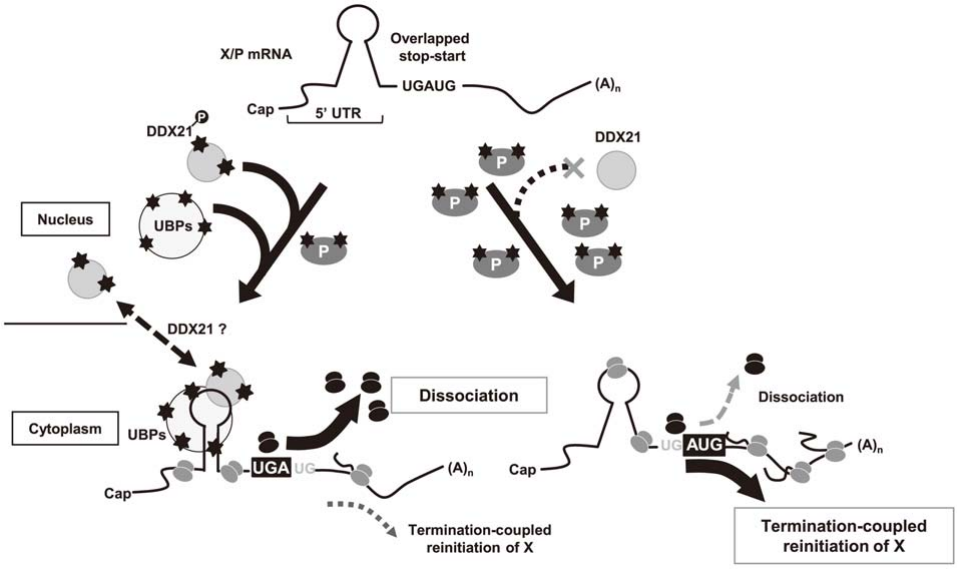
Possible mechanism of autogenous regulation of BDV polycistronic mRNA translation. During the early stage of BDV replication, the phosphorylated DDX21 and other UBPs, including nucleolin, interact with the 5′ UTR of X/P mRNA (left arrow). The interaction may facilitate dissociation or impede X-AUG recognition of ribosomes at the overlapping stop-start codon, leading to inefficient termination-coupled reinitiation, by ribosomes which have translated the uORF, at the X ORF. The DDX21 may be dissociated from the 5′ UTR in the cytoplasm. BDV P accumulation in BDV-infected cells may interfere with phosphorylation of DDX21 and UBPs (right arrow), resulting in the detachment of the RNA helicase complex from the 5′ UTR. The free 5′ UTR may increase the reinitiation processes of ribosomes at X-AUG.

The regulation of ribosomal movement/stability around the overlapping stop-start codon must be crucial for controlling the translation of the downstream ORF. The scanning ribosomes, which have not dissociated from the mRNA after stalling at the uORF termination codon, may be reutilized efficiently for the reinitiation of translation of the X ORF. In favor of this hypothesis, we found that host nuclear factors influence ribosomal initiation of the X ORF through interaction with the 5′ UTR and identified RNA helicase complexes, mainly involving DDX21. DDX21 is a DEAD-box RNA helicase that localizes to the nucleoli and is involved in ribosomal RNA synthesis or processing [38],[52],[53]. Although detailed functions of DDX21 have not been elucidated yet, this helicase appears to fold or unwind RNA or ribonucleoprotein structures through regulation of RNA-RNA or RNA-protein interaction [38],[52],[53]. We found that DDX21 may be a scaffold protein that interacts with the X/P UTR, among the UBPs, and causes structural alteration of the 5′ UTR. Numerous reports have demonstrated that RNA secondary structure contributes to translational control by affecting the constancy of ribosomal scanning on mRNAs or the recognition of initiation signals [6],[54]. The ribosomes may stack or pass through the initiation codons if secondary structures are formed around the initiation site, leading to enhancement or reduction of the translation efficiency of the ORFs. Therefore, it is conceivable that structural modification of the X/P UTR by DDX21 and the UBPs decreases the ribosomal reinitiation at the X ORF or increases ribosomal dissociation from the mRNA after termination of translation of the uORF, both resulting in the suppression of the translation of the X ORF (Figure 10, left arrow). We found that the structural alterations induced by the base-pair changes in a short stem-loop structure within the X/P UTR influence the translation of the X ORF (Figure S8), supporting this conclusion. On the other hand, in this model the structural change of the X/P UTR should occur in the cytoplasm. Considering DDX21 is mostly a nuclear protein [53], it is possible that the transient interaction of DDX21 with X/P mRNA in the nucleus is enough to maintain the structure of X/P UTR in the cytoplasm by introducing the UBPs (Figure 10, left arrow). Alternatively, DDX21 may be transported to the cytoplasm along with the mRNA in this case.

We revealed that phosphorylation of DDX21, as well as nucleolin, is inhibited by expression of P. Previous studies demonstrated that hyperphosphorylation of nucleolin increases its RNA binding affinity, whereas dephosphorylation reduces the affinity [42]. In this study, the RNA-binding activity of DDX21 was shown to be reduced significantly in cells expressing P. These data suggested that accumulation of P in infected cells blocks interaction of DDX21 with the X/P UTR, resulting in upregulation of translation of the X ORF by promotion of ribosomal reinitiation (Figure 10, right arrow). Note that in Figure 9D the P^S26/28A^ did not fully recover the binding activity of DDX21 to the X/P UTR. This suggests that hypophosphorylation of DDX21 may be not exclusively involved in the promotion of the translation of X, although the *in vitro* binding assay based on the transfection may be insensitive for the detection of the binding activity of DDX21 to the 5′ UTR.

Previous studies showed that the intranuclear stoichiometry of N and P is important for BDV polymerase activity and that accumulation of P in the nucleus markedly disturbs both viral replication and persistent infection [21],[55],[56]. Interestingly, it has been demonstrated that X binds directly to P and promotes translocation of P to the cytoplasm from the nucleus [30],[33]. Therefore, P-dependent translational regulation of X must be a convenient and effective mechanism for ensuring an optimal level of P in the nucleus. The nuclear accumulation of P above the threshold level upregulates the translation of X, thereby leading to the translocation of P to the cytoplasm. This could keep the amount of P at the optimal level in the nucleus, which is unequivocally necessary for productive replication and/or persistent infection of BDV in the nucleus. A previous study, which demonstrated that the mutations in Ser26 and Ser28 of P aberrantly upregulate the viral polymerase complex activity, and that recombinant BDVs containing the phosphorylation mutations (rBDV-P^S26/28A^) reduce the expression of X in infected cells [43], may be consistent with our findings, although the possibility that two amino acid changes inevitably introduced in the X ORF of rBDV-P^S26/28A^ affect the expression level of X has remained. In addition, a recent work using a mutant rBDV, which ectopically expresses X under the different transcriptional unit, demonstrated that the expression of X from the mutant virus is not as tightly linked to expression of P as in the wild type BDV, resulting in strong attenuation of the replication of the rBDV in rat brains [57]. This observation may also support our conclusion that the X/P UTR is not only specifically involved in the regulational expression of X but also essentially controls the balanced expression between X and P in infected cells. Furthermore, a recent work by Poenisch et al. [31] showed that recombinant BDVs containing either a premature stop codon in the uORF or mutations ablating the stop codons of the uORF express wild-type like X and P in cultured cells and efficiently replicate in the brains of adult rats. Although this observation may seem to conflict with our findings that the overlapped termination of uORF is critical for the translation reinitiation of X, the recombinant viruses may be able to recover the translation level of X by the expression of other transcription unit, such as a 1.9-kb mRNA, resulting in the efficient replication in infected cells. In fact, Poenisch et al. [31] have demonstrated that the 1.9-kb mRNA not only serves as a template for the synthesis of N but also might be used for the translation of the viral P protein and possibly X, suggesting that the 1.9-kb transcript is a multicistronic mRNA of BDV.

This is the first example, to our knowledge, of autogenous translational regulation of polycistronic mRNA mediated by its own encoding protein and host RNA helicases. The detailed description of the mechanism should provide novel insights into not only an ingenious strategy of virus replication but also the roles of RNA helicases in the translation of eukaryotic mRNAs. Further study remains to be done to discover cellular mRNAs using a similar translation strategy.

## Materials and Methods

### Cell culture and virus

The COS-7 cell line was grown in Dulbecco's modified Eagle's medium (DMEM) supplemented with 5% heat-inactivated fetal calf serum (FCS) at 37°C in a humidified atmosphere of 95% air and 5% CO2. The OL cell line, derived from a human oligodendroglioma, was grown in high-glucose (4.5%) DMEM supplemented with 5% FCS. Cells were passaged every 3 days. The BDV strain huP2br [32],[58] was used for analyses in this study.

### Plasmids

Construction of the expression plasmids for BDV X/P mRNA, N, P and P phosphorylation mutants has been described elsewhere [29],[30],[32],[33],[39],[43]. The mutant forms of the plasmids were generated using PCR-based site-directed mutagenesis. To generate X/P-Luciferase hybrid mRNAs, a luciferase gene was fused in frame with the X and P ORFs at the 148 nt and 149 nt positions of the coding sequences, respectively, and introduced into the pcDNA3 vector (Invitrogen) at the *Kpn* Ι-*Not* Ι sites. The first AUG codon of Luc was replaced by AAG. For expression of DDX21, DDX50, nucleolin, TOP1 and hnRNPQ1, corresponding cDNAs were amplified by RT-PCR from OL cells and inserted into pcXN2, pET32a or pET42a vectors (Novagen). Cells were transfected with equimolar ratios of plasmid DNAs using Lipofectamine™ 2000 (Invitrogen) or FuGENE6 (Roche Applied Science), according to the manufacturer's instructions, and cellular samples were collected at the desired times. The introduction of the correct sequences for the wild type and its mutant were confirmed by DNA sequencing and Western blotting analysis of protein production.

To generate glutathione S-transferase (GST)-tagged DDX21 and nucleolin recombinant proteins used in the Escherichia coli system, we cloned the amplified cDNAs into the pET42a vector (Novagen). The vectors were transformed into BL21 (DE3) (Novagen), and the expression of the GST-tagged proteins was induced by the addition of 0.3 mM IPTG. The cell pellets were resuspended in PBS(-) and then lysed by sonication. After centrifugation, the supernatants were loaded on glutathione sepharose 4B (Amersham Biosciences). Eluted proteins were concentrated using Centricon spin columns (Millipore Corporation) and dialyzed against a 20 mM HEPES (pH 7.5)-100 mM KCl buffer.

The His-tagged DDX21 was generated by the insertion of the PCR-based DDX21 cDNA into PET32a vector (Novagen) and the resultant plasmid was transformed into Rosetta-gami B(De3)pLysS competent cells (Novagen). Purification of the recombinant DDX21 using Ni-NTA agarose (QIAGEN) was performed according to the manufacturer's recommendations.

### Luciferase reporter assay

COS-7 and OL cells cultured in 12-well plates were transfected with plasmids expressing X/P-Luc hybrid mRNA. At 6 h post-transfection, cells were lysed and subjected to luciferase assay system (Promega Corporation), according to the manufacturer's recommendations. The relative levels of luciferase activity were calculated for each fusion plasmid.

### Western blot and immunoprecipitation analyses

For Western blotting, equal amounts of total lysate proteins of COS-7 or OL cells transfected with expression plasmids were subjected to SDS-PAGE and transferred onto polyvinylidene difluoride membrane (Millipore Corporation). Antibodies used in this study were as follows: anti-BDV P mouse monoclonal, anti-BDV X rabbit polyclonal antibodies [30],[33], mouse anti-Flag M2 (Sigma-Aldrich), mouse anti-HA 12CA5 (Roche Applied Science), rabbit DDX21 (Bethyl Laboratories), rabbit Nucleolin (Novus Biologicals), rabbit anti-Topo1 (TopGEN, Inc), mouse hnRNP-Q (ImmunoQuest Ltd), rabbit anti-phosphoserine (ZYMED Laboratories).

For immunoprecipitation (IP) assay, OL cells transfected with Flag-tagged plasmids were lysed with RIPA buffer [20 mM Tris-HCl (pH 7.4), 150 mM NaCl, 2 mM EDTA, 1% Nonidet P-40 (NP-40), 1% Na-deoxycholate with protease inhibitors]. After centrifugation at 15,000 rpm for 30 min, the supernatants were incubated with 40 µl of pre-equilibrated anti-Flag M2 agarose (Sigma-Aldrich) overnight at 4°C with gentle rotation. After incubation, beads were collected by centrifugation at 6,000 rpm for 40 s and washed four times with 1 ml of RIPA. The proteins immunoprecipitated with anti-Flag agarose were eluted with 3×Flag peptide (Sigma-Aldrich) in RIPA buffer and detected by Western blotting as described above. In IP for detection of phosphoserine, NaF and Na3VO4 were added in RIPA buffer, and the serine-phosphorylated proteins were detected by anti-phosphoserine antibody.

### IP-RT-PCR

To detect the interaction of host factors with BDV X/P mRNA *in vivo*, BDV-infected OL cells were transfected with Flag-tagged targeted proteins and lysed with RIPA buffer with RNasin (Promega). After IP with anti-Flag M2, the co-immunoprecipitants were boiled in TE buffer and then treated with RNase-free DNase Ι for 20 min. Total RNAs were isolated from the aqueous solution and used as templates for RT-PCR using specific primers of X/P mRNA.

### *In vitro* translation assay

*In vitro* transcribed X/P-Luc mRNAs were prepared with Maxiscript Kits (Ambion). About 1.0 pmol of X/P-Luc mRNAs were pre-incubated with nuclear extracts of OL cells (total protein 1 to 4 µg) in a total 20 µl of binding mixture [10 mM HEPES (pH 7.6), 67 mM NaCl, 2 mM MgCl_2_, 1 mM DTT, 1 mM EDTA, 5% glycerol, 10U RNasin] for 30 min at room temperature. For competition, a serial dilutions of decoy RNAs were pre-incubated with the extracts prior to the reaction. Binding mixtures were then subjected to the *in vitro* translation system using 50 µl of rabbit reticulocyte lysate (Promega), according to the manufacturer's recommendations. After incubation period of 2 h at 30°C, 10 µl of mixture was subjected to luciferase assay as described above.

### RNA EMSA

The ^32^P labeled-transcripts corresponding to the X/P and M/G UTRs were prepared with a mirVana miRNA Probe construction kit (Ambion), using PCR products or synthetic oligonucleotides as dsDNA templates. Transcription of the X/P and M/G UTRs was confirmed by their mobility in native PAGE. Unlabeled transcripts were prepared with MEGAshortscript™ T7 Kit (Ambion). The cell extracts were obtained from exponentially growing OL cells. The cells were lysed with buffer A [20 mM HEPES (pH 7.6), 10 mM NaCl, 1.5 mM MgCl_2_, 0.2 mM EDTA, 1 mM DTT, 0.1% NP-40, 20% glycerol and protease inhibitor cocktail] and then incubated on ice for 5 min. After collection of the cells, the lysate was incubated for a further 10 min. After centrifugation at 2,000 rpm for 5 min, the supernatant was collected as the cytoplasmic extract. The pellet was lysed with buffer B [20 mM HEPES (pH 7.6), 500 mM NaCl, 1.5 mM MgCl_2_, 0.2 mM EDTA, 1 mM DTT, 0.1% NP-40, 20% glycerol and protease inhibitor cocktail], incubated on ice for 30 min and separated by centrifugation at 15,000 rpm for 15 min. The soluble nuclear fraction was diluted in binding buffer [10 mM HEPES (pH 7.6), 100 mM NaCl, 1.5 mM MgCl_2_, 1 mM EDTA, 1 mM DTT, 0.1% NP-40, 10% glycerol]. About 1.0 pmol of ^32^P-labeled gel-purified probes was incubated with the nuclear extracts (4 µg) in a total of 30 µl of binding mixture [10 mM HEPES (pH 7.6), 67 mM NaCl, 2 mM MgCl_2_, 1 mM DTT, 1 mM EDTA, 5% glycerol, 20 µg tRNA, 10 U RNasin] for 20 min at room temperature. For competition, non-labeled probes were incubated with the nuclear extract for 20 min at room temperature prior to incubation with the labeled probes. For the assays using recombinant proteins, the probes were incubated with 5 pmol of GST-tagged DDX21 or nucleolin in a total of 20 µl of binding mixture [20 mM HEPES (pH7.5), 70 mM KCl, 2 mM MgCl_2_, 2 mM DTT, 0.2 mg/ml BSA, 20 U RNasin] for 10 min at 30°C and for 10 min at room temperature. The reaction mixtures were applied to 4% native polyacrylamide gels (40∶1 acrylamide-bisacrylamide) in TBE buffer. After electrophoresis, the gels were exposed to X-ray film overnight at −80°C.

### RNA-affinity column purification of the 5′ UTR-binding proteins

Nuclear extracts of OL cells were prepared as described above. The nuclear extracts were passed through the RNA-negative coupled column and then loaded onto a consecutive RNA-positive column to remove nonspecific binding proteins. The extracts (total 2.5 mg of protein) were loaded on HiTrap Streptavidin HP column (1.0 ml bed volume; GE Healthcare) equilibrated with binding buffer three times (0.2 ml/min). The flow-through was incubated with 0.02 µmol of a 5′-biotinylated short (20 mer) RNA probe in binding buffer on ice for 30 min, and passed through a HiTrap column three times (0.2 ml/min). The column was washed with 30 ml binding buffer and then the proteins were eluted from the columns by the addition of binding buffer containing 600 mM NaCl. After dialysis with binding buffer, the sample was subjected to an X/P UTR- or M/G UTR-coupled column as a second step of RNA-affinity purification. After washing, the binding proteins were eluted from the column with the same as for the short RNA probe-coupled column.

### LC-MS/MS

Samples eluted from the RNA affinity columns were separated on 10% SDS-PAGE and visualized by silver-staining (Wako). The protein bands of interest were excised, digested in-gel with trypsin, and analyzed by nanocapillary reversed-phase LC-MS/MS using a C18 column (ϕ 75 µm) on a nanoLC system (Ultimate, LC Packing) coupled to a quadrupole time-of-flight mass spectrometer (QTOF Ultima, Waters). Direct injection data-dependent acquisition was performed using one MS channel for every three MS/MS channels and dynamic exclusion for selected ions. Proteins were identified by database searching using Mascot Server (Matrix Science).

### GST-pull down assay

For the protein pull-down assay, 200 pmol of recombinant His-DDX21 and approximately 100 pmol of truncated forms of GST-nucleolin were incubated with RIPA buffer for 1 h at 4°C. After the incubation, reaction mixtures were bound to glutathione-Sepharose 4B (Amersham Biosciences) in RIPA buffer overnight at 4°C. After washing with the same buffer three times, bound proteins were analyzed by immunoblotting with anti-DDX21 antibodies.

### *In vitro* RNA folding assay

About 1.0 pmol of ^32^P-labeled probes were heated at 85°C for 5 min, quickly cooled on ice and equilibrated at 23°C for 20 min prior to the reaction, unless manipulated further. These RNAs were incubated with 5 pmol of GST-tagged DDX21 and GST-tagged truncated nucleolin, Nuc(1234R) in total 15 µl of binding mixture [20mM HEPES (pH 7.5), 70 mM KCl, 3 mM ATP, 0.2 mg/ml BSA, 20 U RNasin] at 23°C for 20 min. After the incubation, the reaction was terminated by the addition of 5×loading buffer [20 mM HEPES (pH 7.5), 70 mM KCl, 50% glycerol, 0.5% SDS, 0.2 mg/ml proteinase K, 0.01% BPB, 0.01% XC], which also inactivated the enzyme. A part of the reaction mixtures was then applied to 12% native polyacrylamide gel (40∶1 acrylamide-bisacrylamide) in TBE buffer. After electrophoresis, the gels were exposed to X-ray film overnight at −80°C.

### *In vitro* RNA binding assay

The OL cells expressing Flag-tagged recombinant proteins were lysed with RIPA buffer including protease inhibitors and 40 µg/ml of RNase A, and IP were performed using anti-Flag M2 as described above. The precipitants were washed twice with washing buffer [20 mM Tris-HCl (pH 7.5), 70 mM NaCl, 70 mM KCl, 0.1% NP-40] and once with binding buffer [20 mM Tris-HCl (pH 7.5), 70 mM KCl, 0.1% NP-40] and subjected to *in vitro* binding assay. 10 pmol of ^32^P-labeled X/P UTR probe was added to 20 µl of 50% suspension of the protein-loaded beads. After adjusting the total volume to 250 µl with binding buffer, the reaction mixture was incubated at 4°C for 10 min with gentle agitation. Unbound probe was removed by washing three times with 1 ml of binding buffer. The amount of bound radio-activity was measured by scintillation counting and the specificity was achieved by eliminating background activity obtained from the bead with the mock-transfected cell extract.

## Supporting Information

Figure S1 The translation of X is suppressed at an early stage of BDV infection. BDV strain He80 was infected into C6 (rat glioma) or OL (human oligodendroglioma) cells. The subcellular localization of X and P was determined by immunofluorescence assay using anti-BDV P and X antibodies. The cells were analyzed when the infection rate was below 5% and reached 100% as early and persistent stages, respectively.
(0.54 MB PDF)

Figure S2 The translation of uORF influences translation of the X ORF. (A) Schematic representation of deletion mutants of the 5′ UTR of X/P expression plasmid. The nucleotide regions deleted from the wt plasmid are shown. The nucleotide region between 23 and 42 contains a short-stem loop structure shown in Figure S8. (B) OL cells were transfected with 0.8 µg of each plasmid and at 12 h after transfection cells were harvested and subjected to Western blotting using anti-BDV P and X antibodies. (C) Fold-activation of X expression in the cells transfected with mutant plasmids was determined after quantitation of band intensities by ImageJ software.
(0.14 MB PDF)

Figure S3 Premature termination of uORF affects the expression of X. (A) Structure of uORF mutants. The nucleotide sequences substituted from the wt plasmid are indicated. These mutations do not induce structural modification of the 5′ UTR of X/P mRNA. (B) Expression of BDV P and X from the mutant uORF expression plasmids. OL cells cultured in 12 well culture dishes were transfected with 0.8 µg of wt and uORF mutant plasmids. Twelve h post-transfection, cells were lysed and subjected to western blot analysis using anti-BDV P and X antibodies. (C) Relative expression of X and P in uORF mutant plasmid-transfected OL cells. The band intensities shown in (B) were determined after quantitation by ImageJ software. The means plus S.D. of three independent experiments are shown. **P<0.01, (Student's t test).
(0.17 MB PDF)

Figure S4 Additional 5′ or Kozak's stem-loop structures in the 5′ UTR inhibit translation initiation of X and P. (A and B) Expression of BDV P and X from the 5′ UTR mutant plasmids. Schematic structure of 5′ UTR mutants is shown. The 5′-stem and Kozak-stem were introduced upstream of the uAUG and by replacing with uORF coding sequence, respectively. OL cells cultured in 12-well culture dishes were transfected with 0.8 µg of each plasmid. Forty-eight h post-transfection, cells were lysed and subjected to western blot analysis using anti-BDV P and X antibodies. (C) Nucleotide sequences of artificial stem structures.
(0.17 MB PDF)

Figure S5 The predicted peptide of uORF does not influence translation of X ORF. (A) Structure of a uORF mutant. The nucleotide and amino acid sequences substituted from the wt plasmid are indicated by black squares. These mutations do not induce structural modification of the 5′ UTR of X/P mRNA. 1 (wt): wild-type uORF, 2: mutant uORF. (B) Expression of BDV P and X from the mutant uORF expression plasmid. OL cells cultured in 12-well culture dishes were transfected with 0.8 µg of wt and uORF mutant plasmids. Forty-eight h post-transfection, cells were lysed and subjected to western blot analysis using anti-BDV P and X antibodies. (C) Relative expression of X and P in uORF mutant plasmid-transfected OL cells. The band intensities shown in (B) were determined after quantitation by ImageJ software. The means plus S.D. of three independent experiments are shown.
(0.12 MB PDF)

Figure S6 BDV P does not affect the functions of eukaryotic initiation factors. (A) OL cells were transfected with Flag-tagged BDV N or P plasmid and, forty-eight h post-transfection, cells were lysed with RIPA or TNE buffer and then immunoprecipitated with Flag-M2 affinity gel. Immunoprecipitates were analyzed using the indicated antibodies. CE indicates cell extract. (B) Expression and phosphorylation of eIFs in BDV P-expressed cells. The cells expressing BDV N or P were analyzed by western blotting using the antibodies indicated. The phosphorylation of eIF2α was detected by phosphoserine 51-eIF2α antibody.
(0.26 MB PDF)

Figure S7 GST-pull down assay of recombinant nucleolin. (A) Schematic representation of truncation mutants of recombinant GST-fused nucleolin. (B) *In vitro* pull-down assay between His-tagged DDX21 and GST-fused recombinant nucleolins. 200 pmol of recombinant His-DDX21 and approximately 100 pmol of truncated GST-fused nucleolins were incubated with RIPA buffer for 1 h at 4°C. Proteins precipitated with glutathione-Sepharose beads were immunoblotted with anti-DDX21 antibody. Coomassie brilliant blue (CBB) staining of His-tagged DDX21 and GST-fused nucleolins at the top, which were bacterially expressed, purified, and used for *in vitro* binding.
(0.14 MB PDF)

Figure S8 The stability of the 5′ UTR controls translation of X ORF. (A) Schematic representation of a short stem-loop (SL) structure in the 5′ UTR. (B) Base-pair changing mutations were introduced within the SL region. The nucleotide substitutions are indicated by black squares. OL cells were transfected with 0.8 µg of wt and SL mutants, and at 48 h post-transfection, cells were subjected to western blotting using anti-BDV P mouse monoclonal and anti-BDV X rabbit polyclonal antibodies. Predicted free energies (kcal/mol) of SL structures are shown. (C) Relative expression of X and P was determined after quantitation of band intensities by ImageJ software. The mean plus S.D. of three independent experiments are shown.
(0.12 MB PDF)
