# Supplementary material for: Autogenous Translational Regulation of the Borna Disease Virus Negative Control Factor X from Polycistronic mRNA Using Host RNA Helicases
Source: PLoS Pathog. 2009 Nov 6;5(11):e1000654. doi: 10.1371/journal.ppat.1000654 (PMC2766071; doi:10.1371/journal.ppat.1000654)
Supplement: Figure S1 — The translation of X is suppressed at an early stage of BDV infection. BDV strain He80 was infected into C6 (rat glioma) or OL (human oligodendroglioma) cells. The subcellular localization of X and P was determined by immunofluorescence assay using anti-BDV P and X antibodies. The cells were analyzed when the infection rate was below 5% and reached 100% as early and persistent stages, respectively. (0.54 MB PDF) [file ppat.1000654.s001.pdf]

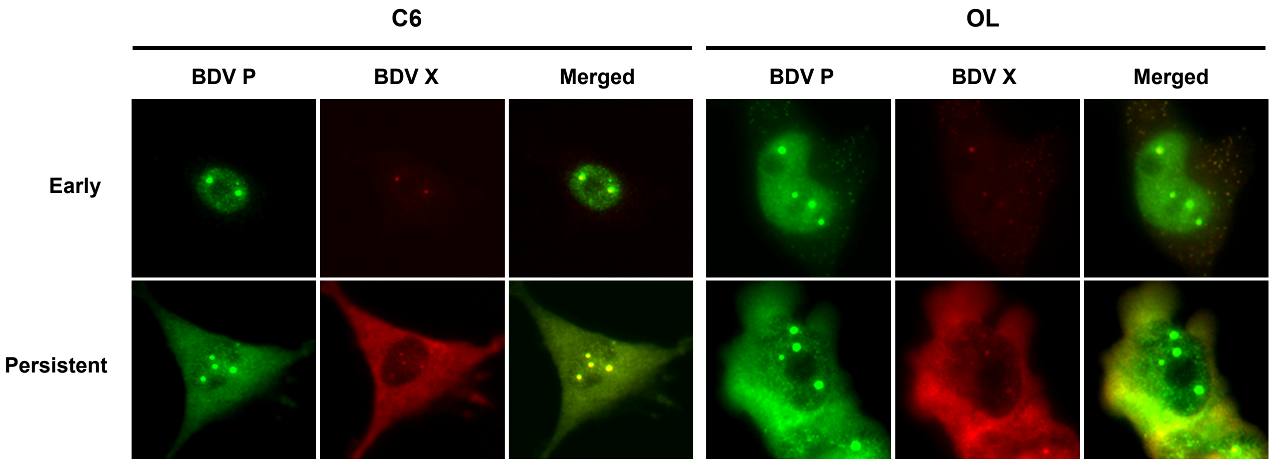

Figure S1

The translation of X is suppressed at an early stage of BDV infection. BDV strain He80 was infected into C6 (rat glioma) or OL (human oligodendroglioma) cells. The subcellular localization of X and P was determined by immunofluorescence assay using anti-BDV P and X antibodies. The cells were analyzed when the infection rate was below 5% and reached 100% as early and persistent stages, respectively.
