# Supplementary material for: Autogenous Translational Regulation of the Borna Disease Virus Negative Control Factor X from Polycistronic mRNA Using Host RNA Helicases
Source: PLoS Pathog. 2009 Nov 6;5(11):e1000654. doi: 10.1371/journal.ppat.1000654 (PMC2766071; doi:10.1371/journal.ppat.1000654)
Supplement: Figure S2 — The translation of uORF influences translation of the X ORF. (A) Schematic representation of deletion mutants of the 5′ UTR of X/P expression plasmid. The nucleotide regions deleted from the wt plasmid are shown. The nucleotide region between 23 and 42 contains a short-stem loop structure shown in Figure S8. (B) OL cells were transfected with 0.8 µg of each plasmid and at 12 h after transfection cells were harvested and subjected to Western blotting using anti-BDV P and X antibodies. (C) Fold-activation of X expression in the cells transfected with mutant plasmids was determined after quantitation of band intensities by ImageJ software. (0.14 MB PDF) [file ppat.1000654.s002.pdf]

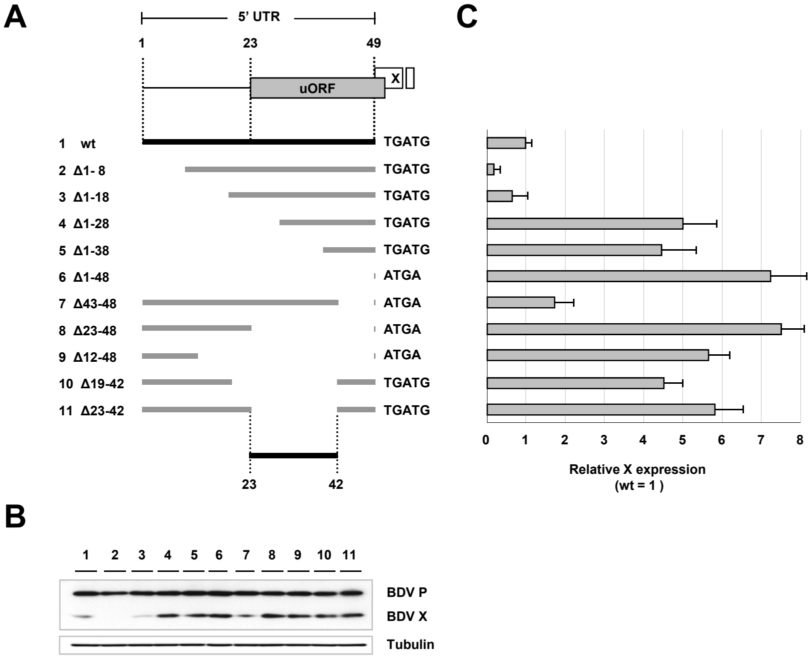

Figure S2

The translation of uORF influences translation of the X ORF.

(**A**) Schematic representation of deletion mutants of the 5' UTR of X/P expression plasmid. The nucleotide regions deleted from the wt plasmid are shown. The nucleotide region between 23 and 42 contains a short-stem loop structure shown in Figure S8. (**B**) OL cells were transfected with 0.8 μg of each plasmid and at 12 h after transfection cells were harvested and subjected to Western blotting using anti-BDV P and X antibodies. (**C**) Fold-activation of X expression in the cells transfected with mutant plasmids was determined after quantitation of band intensities by ImageJ software.
