# Supplementary material for: Autogenous Translational Regulation of the Borna Disease Virus Negative Control Factor X from Polycistronic mRNA Using Host RNA Helicases
Source: PLoS Pathog. 2009 Nov 6;5(11):e1000654. doi: 10.1371/journal.ppat.1000654 (PMC2766071; doi:10.1371/journal.ppat.1000654)
Supplement: Figure S3 — Premature termination of uORF affects the expression of X. (A) Structure of uORF mutants. The nucleotide sequences substituted from the wt plasmid are indicated. These mutations do not induce structural modification of the 5′ UTR of X/P mRNA. (B) Expression of BDV P and X from the mutant uORF expression plasmids. OL cells cultured in 12 well culture dishes were transfected with 0.8 µg of wt and uORF mutant plasmids. Twelve h post-transfection, cells were lysed and subjected to western blot analysis using anti-BDV P and X antibodies. (C) Relative expression of X and P in uORF mutant plasmid-transfected OL cells. The band intensities shown in (B) were determined after quantitation by ImageJ software. The means plus S.D. of three independent experiments are shown. **P<0.01, (Student's t test). (0.17 MB PDF) [file ppat.1000654.s003.pdf]

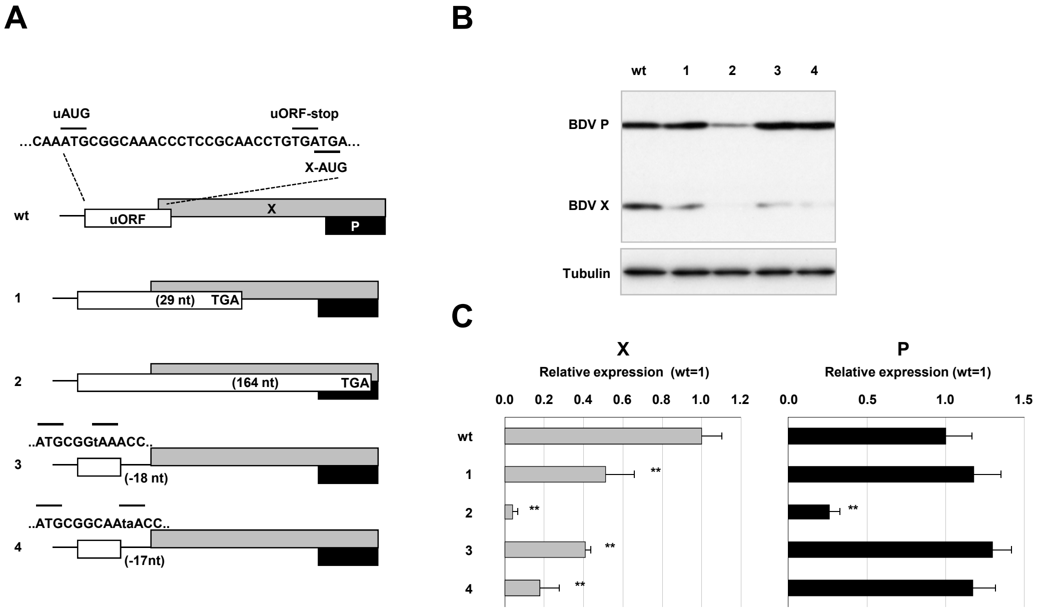

Figure S3

Premature termination of uORF affects the expression of X.
(**A**) Structure of uORF mutants. The nucleotide sequences substituted from the wt plasmid are indicated. These mutations do not induce structural modification of the 5' UTR of X/P mRNA. (**B**) Expression of BDV P and X from the mutant uORF expression plasmids. OL cells cultured in 12 well culture dishes were transfected with 0.8 μg of wt and uORF mutant plasmids. Twelve h post-transfection, cells were lysed and subjected to western blot analysis using anti-BDV P and X antibodies. (**C**) Relative expression of X and P in uORF mutant plasmid-transfected OL cells. The band intensities shown in (B) were determined after quantitation by ImageJ software. The means plus S.D. of three independent experiments are shown. $**P <$ 0.01, (Student's t test).
