# Supplementary material for: Autogenous Translational Regulation of the Borna Disease Virus Negative Control Factor X from Polycistronic mRNA Using Host RNA Helicases
Source: PLoS Pathog. 2009 Nov 6;5(11):e1000654. doi: 10.1371/journal.ppat.1000654 (PMC2766071; doi:10.1371/journal.ppat.1000654)
Supplement: Figure S4 — Additional 5′ or Kozak's stem-loop structures in the 5′ UTR inhibit translation initiation of X and P. (A and B) Expression of BDV P and X from the 5′ UTR mutant plasmids. Schematic structure of 5′ UTR mutants is shown. The 5′-stem and Kozak-stem were introduced upstream of the uAUG and by replacing with uORF coding sequence, respectively. OL cells cultured in 12-well culture dishes were transfected with 0.8 µg of each plasmid. Forty-eight h post-transfection, cells were lysed and subjected to western blot analysis using anti-BDV P and X antibodies. (C) Nucleotide sequences of artificial stem structures. (0.17 MB PDF) [file ppat.1000654.s004.pdf]

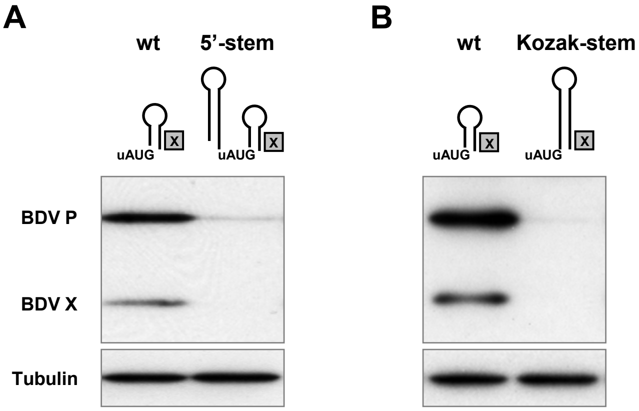

C

5’-stem: TTGATTAGATCTGGTACCGAGCTCCCCGGGCTGCAGCCCGGGGAGCTCGGTACCCAGATCTAA

Kozak-stem: atGGGGGCGCGTGGTGGCGGCTGCAGCCGCCACCACGCGCCCCCaa

uAUG

Figure S4

Additional 5’ or Kozak’s stem-loop structures in the 5’ UTR inhibit translation initiation of X and P.

(**A and B**) Expression of BDV P and X from the 5’ UTR mutant plasmids. Schematic structure of 5’ UTR mutants is shown. The 5’-stem and Kozak-stem were introduced upstream of the uAUG and by replacing with uORF coding sequence, respectively. OL cells cultured in 12-well culture dishes were transfected with 0.8 μg of each plasmid. Forty-eight h post-transfection, cells were lysed and subjected to western blot analysis using anti-BDV P and X antibodies. (**C**) Nucleotide sequences of artificial stem structures.
