# Supplementary material for: Autogenous Translational Regulation of the Borna Disease Virus Negative Control Factor X from Polycistronic mRNA Using Host RNA Helicases
Source: PLoS Pathog. 2009 Nov 6;5(11):e1000654. doi: 10.1371/journal.ppat.1000654 (PMC2766071; doi:10.1371/journal.ppat.1000654)
Supplement: Figure S5 — The predicted peptide of uORF does not influence translation of X ORF. (A) Structure of a uORF mutant. The nucleotide and amino acid sequences substituted from the wt plasmid are indicated by black squares. These mutations do not induce structural modification of the 5′ UTR of X/P mRNA. 1 (wt): wild-type uORF, 2: mutant uORF. (B) Expression of BDV P and X from the mutant uORF expression plasmid. OL cells cultured in 12-well culture dishes were transfected with 0.8 µg of wt and uORF mutant plasmids. Forty-eight h post-transfection, cells were lysed and subjected to western blot analysis using anti-BDV P and X antibodies. (C) Relative expression of X and P in uORF mutant plasmid-transfected OL cells. The band intensities shown in (B) were determined after quantitation by ImageJ software. The means plus S.D. of three independent experiments are shown. (0.12 MB PDF) [file ppat.1000654.s005.pdf]

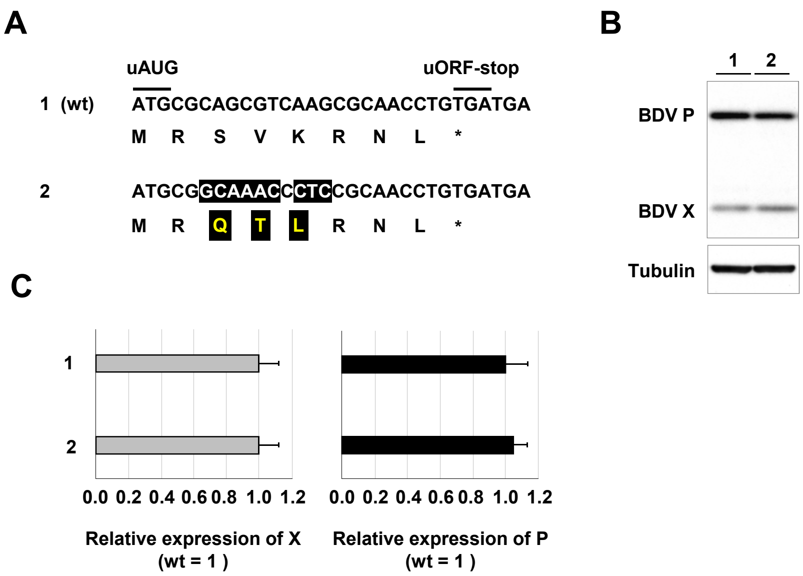

Figure S5

The predicted peptide of uORF does not influence translation of X ORF.
(**A**) Structure of a uORF mutant. The nucleotide and amino acid sequences substituted from the wt plasmid are indicated by black squares. These mutations do not induce structural modification of the 5' UTR of X/P mRNA. 1 (wt): wild-type uORF, 2: mutant uORF. (**B**) Expression of BDV P and X from the mutant uORF expression plasmid. OL cells cultured in 12-well culture dishes were transfected with 0.8 μg of wt and uORF mutant plasmids. Forty-eight h post-transfection, cells were lysed and subjected to western blot analysis using anti-BDV P and X antibodies. (**C**) Relative expression of X and P in uORF mutant plasmid-transfected OL cells. The band intensities shown in (B) were determined after quantitation by ImageJ software. The means plus S.D. of three independent experiments are shown.
