# Supplementary material for: Autogenous Translational Regulation of the Borna Disease Virus Negative Control Factor X from Polycistronic mRNA Using Host RNA Helicases
Source: PLoS Pathog. 2009 Nov 6;5(11):e1000654. doi: 10.1371/journal.ppat.1000654 (PMC2766071; doi:10.1371/journal.ppat.1000654)
Supplement: Figure S6 — BDV P does not affect the functions of eukaryotic initiation factors. (A) OL cells were transfected with Flag-tagged BDV N or P plasmid and, forty-eight h post-transfection, cells were lysed with RIPA or TNE buffer and then immunoprecipitated with Flag-M2 affinity gel. Immunoprecipitates were analyzed using the indicated antibodies. CE indicates cell extract. (B) Expression and phosphorylation of eIFs in BDV P-expressed cells. The cells expressing BDV N or P were analyzed by western blotting using the antibodies indicated. The phosphorylation of eIF2α was detected by phosphoserine 51-eIF2α antibody. (0.26 MB PDF) [file ppat.1000654.s006.pdf]

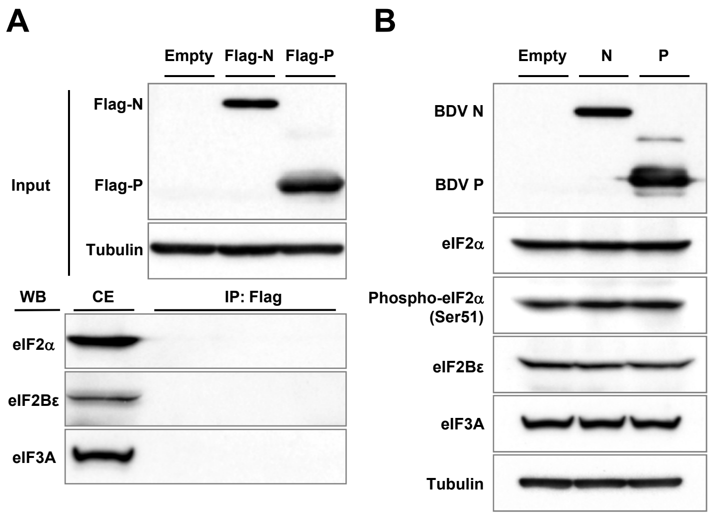

Figure S6

BDV P does not affect the functions of eukaryotic initiation factors.
(**A**) OL cells were transfected with Flag-tagged BDV N or P plasmid and, forty-eight h post-transfection, cells were lysed with RIPA or TNE buffer and then immunoprecipitated with Flag-M2 affinity gel. Immunoprecipitates were analyzed using the indicated antibodies. CE indicates cell extract. (**B**) Expression and phosphorylation of eIFs in BDV P-expressed cells. The cells expressing BDV N or P were analyzed by western blotting using the antibodies indicated. The phosphorylation of eIF2α was detected by phosphoserine 51-eIF2α antibody.
