# Supplementary material for: Autogenous Translational Regulation of the Borna Disease Virus Negative Control Factor X from Polycistronic mRNA Using Host RNA Helicases
Source: PLoS Pathog. 2009 Nov 6;5(11):e1000654. doi: 10.1371/journal.ppat.1000654 (PMC2766071; doi:10.1371/journal.ppat.1000654)
Supplement: Figure S7 — GST-pull down assay of recombinant nucleolin. (A) Schematic representation of truncation mutants of recombinant GST-fused nucleolin. (B) In vitro pull-down assay between His-tagged DDX21 and GST-fused recombinant nucleolins. 200 pmol of recombinant His-DDX21 and approximately 100 pmol of truncated GST-fused nucleolins were incubated with RIPA buffer for 1 h at 4°C. Proteins precipitated with glutathione-Sepharose beads were immunoblotted with anti-DDX21 antibody. Coomassie brilliant blue (CBB) staining of His-tagged DDX21 and GST-fused nucleolins at the top, which were bacterially expressed, purified, and used for in vitro binding. (0.14 MB PDF) [file ppat.1000654.s007.pdf]

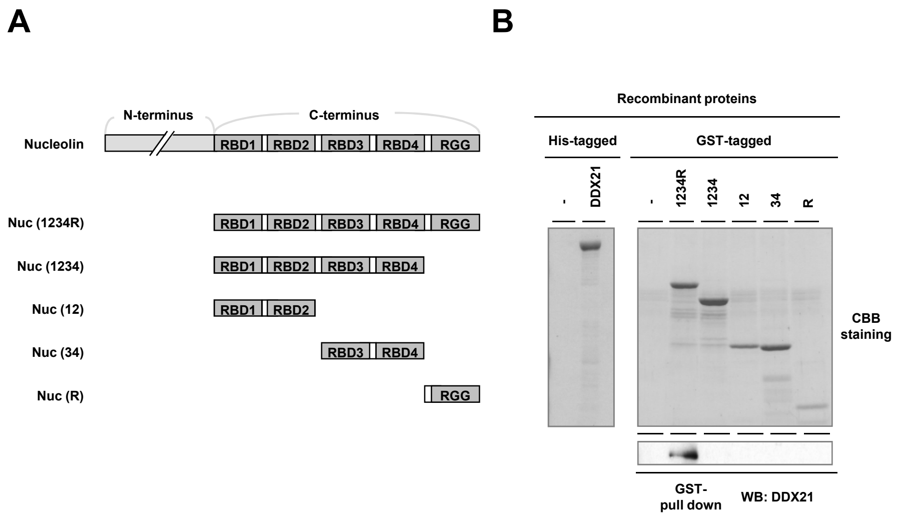

Figure S7

GST-pull down assay of recombinant nucleolin.

(**A**) Schematic representation of truncation mutants of recombinant GST-fused nucleolin. (**B**) *In vitro* pull-down assay between His-tagged DDX21 and GST-fused recombinant nucleolins. 200 pmol of recombinant His-DDX21 and approximately 100 pmol of truncated GST-fused nucleolins were incubated with RIPA buffer for 1 h at 4ºC. Proteins precipitated with glutathione-Sepharose beads were immunoblotted with anti-DDX21 antibody. Coomassie brilliant blue (CBB) staining of His-tagged DDX21 and GST-fused nucleolins at the top, which were bacterially expressed, purified, and used for *in vitro* binding.
