# Supplementary material for: Autogenous Translational Regulation of the Borna Disease Virus Negative Control Factor X from Polycistronic mRNA Using Host RNA Helicases
Source: PLoS Pathog. 2009 Nov 6;5(11):e1000654. doi: 10.1371/journal.ppat.1000654 (PMC2766071; doi:10.1371/journal.ppat.1000654)
Supplement: Figure S8 — The stability of the 5′ UTR controls translation of X ORF. (A) Schematic representation of a short stem-loop (SL) structure in the 5′ UTR. (B) Base-pair changing mutations were introduced within the SL region. The nucleotide substitutions are indicated by black squares. OL cells were transfected with 0.8 µg of wt and SL mutants, and at 48 h post-transfection, cells were subjected to western blotting using anti-BDV P mouse monoclonal and anti-BDV X rabbit polyclonal antibodies. Predicted free energies (kcal/mol) of SL structures are shown. (C) Relative expression of X and P was determined after quantitation of band intensities by ImageJ software. The mean plus S.D. of three independent experiments are shown. (0.12 MB PDF) [file ppat.1000654.s008.pdf]

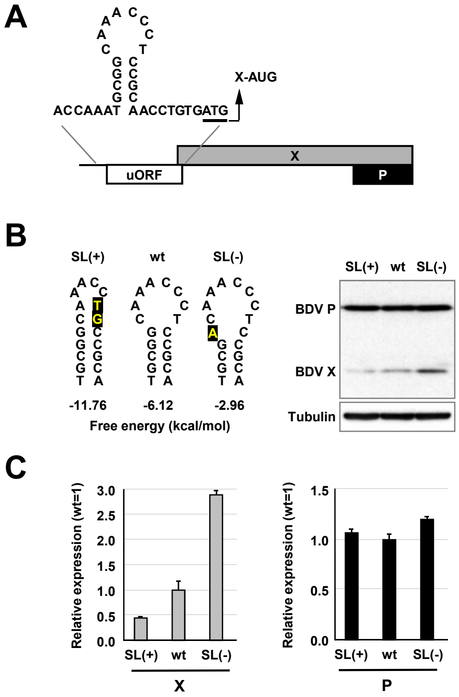

Figure S8

The stability of the 5' UTR controls translation of X ORF.
(**A**) Schematic representation of a short stem-loop (SL) structure in the 5' UTR. (**B**) Base-pair changing mutations were introduced within the SL region. The nucleotide substitutions are indicated by black squares. OL cells were transfected with 0.8 μg of wt and SL mutants, and at 48 h post-transfection, cells were subjected to western blotting using anti-BDV P mouse monoclonal and anti-BDV X rabbit polyclonal antibodies. Predicted free energies (kcal/mol) of SL structures are shown. (**C**) Relative expression of X and P was determined after quantitation of band intensities by ImageJ software. The mean plus S.D. of three independent experiments are shown.
